# Key role of ERβ in estrogenic regulation of neuronal mitochondrial function

**DOI:** 10.1002/alz.086633

**Published:** 2025-01-03

**Authors:** Tian Wang, Zisu Mao, Shuhua Chen, Yuan Shang, John W. McLean, James Brett Stanton, Guoyuan Qi, Fei Yin, Roberta Diaz Brinton

**Affiliations:** ^1^ University of Arizona, Tucson, AZ USA

## Abstract

**Background:**

Estrogen is a master regulator of the bioenergetic system in the female brain, exerting broad control over metabolic processes from glucose transport to glycolysis, mitochondrial respiration, and ATP generation. The loss of estrogen during the perimenopausal transition is associated with decline in brain glucose metabolism and mitochondrial function which can contribute to the two‐fold greater lifetime risk of Alzheimer’s disease in postmenopausal women. While both ERα and ERβ have been reported to mediate E2 regulation of brain bioenergetic function, their cell‐type specific contribution to bioenergetic homeostasis has yet to be elucidated.

**Method:**

Herein, we investigated the role of ERα and ERβ in E2 regulation of neuronal bioenergetics. We developed novel conditional estrogen receptor α and β knock‐down rat models by inserting two loxP sites upstream and downstream of exon 3 of *Esr1 or Esr2* allele using Crispr/Cas9‐mediated genome engineering technique. Our novel ERαlox and ERβlox rat models enable inducible and brain cell‐type and region specific ERα and ERβ knockdown without disrupting normal development of estrogen dependent organs and systems. Using these models, ERα and ERβ dependent regulation on neuronal mitochondrial function was investigated *in vivo* and *in vitro*. Further, unbiased transcriptomic analysis was conducted to delineate ERα and ERβ specific signaling pathways modulating neuronal bioenergetic function.

**Result:**

ERβ significantly regulated E2 activation of neuronal mitochondrial oxidative phosphorylation *in vivo*. Selective knockdown of neuronal ERβ resulted in significant decrease in hippocampal mitochondrial respiration and impaired OXPHOS complex activity. In contrast, ERα knockdown did not impact hippocampal mitochondrial respiration nor mitochondrial OXPHOS complex activity. These results were confirmed in primary embryonic ERα and ERβ knockdown neurons. Mechanistic investigation further revealed ERβ dependent transcriptomic regulation of TCA and OXPHOS genes. Importantly, ERβ knockdown resulted in increased UCP2 expression and decreased AKT and ERK signaling, leading to increased proton leak and decreased mitochondrial respiration.

**Conclusion:**

ERβ exerts a pivotal role in maintaining neuronal mitochondrial function and bioenergetic homeostasis in female brain. Selectively activating ERβ could provide a precision medicine strategy to promote brain function in menopausal women while inhibiting breast tissue proliferation.

